# Treatment outcomes of childhood tuberculosis in Addis Ababa: a five-year retrospective analysis

**DOI:** 10.1186/s12889-016-3193-8

**Published:** 2016-07-21

**Authors:** Genene Tilahun, Solomon Gebre-Selassie

**Affiliations:** Senior Microbiologist, Zewditu Memorial Hospital, Addis Ababa, Ethiopia; Department of Microbiology, Immunology and Parasitology, School of Medicine, College of, Health Sciences, Addis Ababa University, Addis Ababa, Ethiopia

**Keywords:** Children, Tuberculosis, Treatment outcome, HIV

## Abstract

**Background:**

Tuberculosis (TB) kills one child every 5 min. Childhood TB is given low priority in most national health programmes particularly in TB-endemic areas. TB among children is an indicator of a recent transmission of the disease in the community. Treatment outcome results serve as a proxy of the quality of treatment provided by a health care system. In Ethiopia, data on treatment outcomes of childhood TB are limited. The aim of the study was to determine the treatment outcomes of childhood TB in a hospital setting in Addis Ababa.

**Methods:**

The study was conducted during June to August 2014. The data of 491 children treated for TB in Zewditu Memorial Hospital during a 5 year (2009–2013) was analysed. TB was diagnosed using standard methods. Demographic and clinical data including type of TB, TB-HIV co-infection and treatment outcomes were collected from registry of the TB clinic. Treatment outcome definitions are used according to the World Health Organization.

**Results:**

Of the 491 children, 272(55.4 %) were females, 107(21.8 %) were under 5 year old, 454(92.5 %) of them were new cases. The types of TB were extra-pulmonary tuberculosis (EPTB) 243(49.5 %) and 248(50.5 %) pulmonary tuberculosis (PTB). Of the PTB cases, 42(16.9 %) were sputum smear positive. Of the 291 children tested for HIV, 82(28.2 %) were positive. The overall treatment success rate was 420(85.5 %) and the poor treatment outcome was 71(14.5 %). Of the children with poor treatment outcome, 9(1.8 %) died, 3(0.6 %) defaulted from treatment, 2(0.4 %) were treatment failure and 55(11.2 %) were transferred out. Males and females had similar treatment success rates of 85.8 % and 85.3 %, respectively. Infants under one year had significantly lower treatment success rate of 72.7 % compared to those above 1 years of age of 86.5 % (*P* < 0.001). Treatment success rate ranged from 78.0 to 92.6 % during the study period. Associated factors for treatment outcome were age above 5 years (AOR = 0.59, 95 % CI: 0.62–0.97) and seropositive for HIV infection (AOR = 6.66, 95 % CI: 3.07–14.47).

**Conclusions:**

The treatment success rate in this study is 85.5 %. The outcome of treatment varied with age, and presence of HIV infection. In order to the further improve of treatment success rate, continuous follow up with frequent support of patients during treatment course and strengthen the recording system are strongly recommend.

**Electronic supplementary material:**

The online version of this article (doi:10.1186/s12889-016-3193-8) contains supplementary material, which is available to authorized users.

## Background

Tuberculosis (TB) remains a major global health problem. It is the second leading cause of death from an infectious disease worldwide, after the human immunodeficiency virus (HIV) [1]. Children account for 6 %–10 % of all TB cases worldwide [2]. TB kills one child every 5 minutes. In countries with high burden TB, it may be as high as 20–40 % of all new TB cases. More than 74,000 children die from the disease each year [2]. Of the one million estimated cases of TB in children worldwide, 75 % occur in the 22 high-burden countries [3]. As childhood TB reflects ongoing transmission in the community, children are affected most acutely in areas where adult TB is poorly controlled [4]. Childhood TB is usually acquired from an infectious adult contact [5]. The global TB control strategy has focused predominantly on smear-positive adult cases and not on the usually paucibacillary and smear negative, noncontagious, asymptomatic childhood TB [6]. TB among children is much more prevalent in developing countries due to poor socio-economic conditions, malnutrition, over-crowding, HIV co-infection and high prevalence of TB in adults contacts [7]. Childhood TB remains neglected for various reasons, mainly the difficulty in diagnosing pulmonary tuberculosis (PTB), the unknown outcomes of children with TB, and the belief that childhood TB is not important for TB control [8, 9]. In 2013, the World Health Organization (WHO) developed a roadmap aiming to achieve zero deaths due to childhood TB by 2025 [10].

Treatment outcome results serve as a proxy of the quality of TB treatment provided by a health care system. Treatment success measured by treatment outcome monitoring (TOM) is a key output of any TB control programs [11]. Treatment outcome in all patients should be routinely monitored by the epidemiological surveillance system. This would make it possible to recognize and amend system failures before the incidence and proportion of resistant isolates rise [12]. According to the 2012 WHO report, Ethiopia ranks seventh among the world’s 22 countries with a high TB burden [1]. Hospital data show that TB is the leading cause of morbidity, third cause of hospital admissions after deliveries and malaria, and second cause of death after malaria in Ethiopia [13]. Although scanty reports on treatment outcomes of TB among adults are available, little is known in children in from Ethiopia [14]. Therefore, this study aimed to assess prevalence and treatment outcomes of TB among children on treatment follow up in Zewditu Memorial Hospital, Addis Ababa.

## Methods

### Study design, study site and period

A 5-year (2009–2013) hospital- based retrospective cohort study was done to assess the treatment outcomes of childhood TB in Zewditu Memorial Hospital in Addis Ababa. The hospital is one of the six district hospitals under the Addis Ababa City administration health bureau. It is the largest treatment centre of HIV infection in the country. The hospital receives children and adults referred from the city and all over the country for diagnosis and treatment. Data for the study was extracted from registry of TB cases treated under directly observed treatment- short course (DOTS) strategy during January 1, 2009 to December 31, 2013. The study was conducted during June to August 2014.

### Study population

A total of 491 children diagnosed as having TB of all types registered during 2009–2013 had follow up in the TB clinic were included in the study. TB patients with incomplete data were excluded from the study. All subjects below the age of 15 years with complete sociodemographic and clinical data were illegible for the study.

### Data collection and diagnostic methods

Data were collected on standardized forms that included demographics (age, sex), clinical history, laboratory and radiographic testing, HIV test results, type and category of TB, treatment outcomes. Diagnosis of TB was made according to the recommendations of the Ministry of Health (MoH) of Ethiopia, which was adopted from the WHO [13]. History of contact with TB patient, signs and symptoms suggestive of TB were taken. Patients presenting with cough lasting for more than 2 weeks smear microscopic examination of sputum for bacteriological confirmation was done. Chest x-ray was also taken. Culture for mycobacteria was done particularly for those suspected to have multi drug resistant TB. Patients with at least two positive smears were considered to have smear-positive pulmonary tuberculosis (SPPTB); those with three negative smears were treated with antibiotics and then re-evaluated. Diagnosis of extra pulmonary tuberculosis (EPTB) was based on fine needle aspiration cytology, biochemical analyses of cerebrospinal/pleural/ ascetic fluids or histopathological examination in addition to clinical evidences consistent with active EPTB. Treatment was based on the the guidelines of the country with a full course of anti-TB therapy (DOTs). Treatment of new TB patients consists of a 2-month intensive phase with Isoniazid, Rifampicin, Pyrazinamide and Ethambutol followed by a 4-month continuation phase with Rifampicin and Isoniazid. Outcomes were categorized as favorable (cured/success) and unfavorable (loss to follow-up, died, failure and transfer out).

### Data analysis

Data was entered using Epi-Data version 3.1 and analyzed using computer software SPSS version 20. Descriptive statistical methods were used to generate frequencies of categorical variables and univariate and multivariate logistic regression analysis was used to investigate the effect of selected risk factors on the incidence of treatment success. The univariate (unadjusted) and multivariate (adjusted) logistic regression analysis were used for odds ratios (ORs) and 95 % confidence interval (95 % CI) was done to assess the association between potential risk factors and treatment outcome. *P* value of 0.05 was used as the cut-off point for statistical significance.

### Ethical issues

Ethical clearance was obtained from Department Ethical and Review Committee (DERC) of Microbiology, Immunology and Parasitology (DMIP), College of Health Sciences, Addis Ababa University. Consent was obtained from parents or guardians during examinations in the TB clinic of the hospital. In order to ensure confidentiality, names of study participants were not included in the data sheet. Information obtained from the data of the study participants is kept confidential.

### Definitions of terms

TB cases were defined according to WHO criteria Table 1.

**Table 1:** Definitions of terms for type of TB, Patient category, and Treatment outcome as per NLCP ^a^ guidelines adopted from the WHO [13]

Category	Definition
Type of TB	
Childhood TB	A person aged 0 – 14 years old who was diagnosed with TB and treated for TB disease
Smear-positive pulmonary tuberculosis (SPPTB)	Patient with at least two sputum specimens with sputum positive for acid-fast bacilli (AFB) by microscopy, or a patient with only one sputum specimen with smear positive for AFB by microscopy and chest radiographic abnormalities consistent with active pulmonary TB.
Smear-negative pulmonary tuberculosis (SNPTB)	Patient with symptoms suggestive of TB with at least two sputum specimens which were negative for AFB by microscopy, and with chest radiographic abnormalities consistent with active PTB (including interstitial or miliary abnormal images), or a patient with two sets of at least two sputum specimens taken at least two weeks apart, and which were negative for AFB by microscopy, and radiographic abnormalities consistent with pulmonary TB and lack of clinical response to one week of broad spectrum antibiotic therapy.
Extra-pulmonary tuberculosis (EPTB)	TB of organs other than the lungs, such as lymph nodes, abdomen, genitourinary tract, skin, joints, bones, meninges, etc.
Patient category:	
New case	Patient who has never had treatment for TB before or has been on anti-TB treatment for less than four weeks.
Relapse	Patient who has been declared cure or treatment completed of any form of TB in the past but who reports back to the health service and is found to be acid fast bacilli smear positive or culture positive.
Treatment failure	Patient who, while on treatment remained smear- positive or become again smear-positive at the end of the five month or later after commencing treatment.
Transfer in	Patient who started treatment in one health facility and transferred to the hospital to continue treatment and follow up.
Retreatment case	Patient who has been treated in the past and include: failure, returned after default, relapse cases, and others, *i.e*. patients who were previously treated for TB and declared cured before becoming once again a definite case of pulmonary TB.
Treatment outcome	
Cured	Finished treatment with negative bacteriology result at the end of treatment
Completed treatment	Finished treatment, but without bacteriology result at the end of treatment
Failure	Remaining smear positive at five months despite correct intake of medication
Default to treatment	Patients who interrupted their treatment for two consecutive months or more after registration
Died	Patients who died from TB during the course of treatment
Transferred out	Patients whose treatment results are unknown due to transfer to another health facility
Loss to follow up	Patient who did not start treatment or whose treatment was interrupted for two consecutive months or more
Successfully treated	A patient who completed treatment and cured
Unsatisfactory treatment outcome	Patient who died from TB during the course of treatment, interrupted treatment for two consecutive months or more after registration, patient remaining smear positive at five months despite correct intake of medication and patient whose treatment results are unknown due to transfer to another health facility
Unknown	No treatment details available (e.g., lost patient notes)

^a^NTLCP = National TB and Leprosy control programme

## Results

### Sociodemographic and clinical characteristics of children with tuberculosis

A total of 652 children diagnosed to have TB in Zewditu Memorial Hospital were included in the study. Of these, 161(24.7 %) were excluded because of incomplete data while 491(75.3 %) of the patients treated for different TB types had were illegible and data were analysed. Of the 491children, 219(44.6 %) were males and 272(55.4 %) females with age range from zero to14 years (mean age of 9.0 ± 4.5SD). Of the total children, under 1 year old had the least contribution of 33(6.7 %) of the total. In total, the under five children comprised of 107(21.8 %). Nearly half, 245(49.9 %) were in the age range of 10–14 years. The remaining patients with incomplete data were excluded from the study. Of all the TB cases, 243(49.5 %) were due to EPTB. Of the PTB cases, 206(83.1 %) were SNPTB and 42(16.9 %) were SPPTB cases. Of the Majority of the children, 454(92.5 %) were new cases, while 19(3.9 %) were transferred in, 5(1.0 %) were retreatment (relapse), 3(0.6 %) cases were default and 2(0.4 %) cases were treatment failures. Of the 291 children tested for HIV, 82(28.2 %) of them were positive thus had TB -HIV co-infection (Table 2).

**Table 2:** Demographic and Clinical Characteristics of TB patients (*N* = 491)

Characteristics	Number	Percent
Gender		
Male	219	44.6
Female	272	55.4
Age groups (years)		
< 1	33	6.7
1 – 4	74	15.1
5 – 9	139	28.3
10 – 14	245	49.9
TB category		
Pulmonary (248)		
Smear positive	42	16.9
Smear negative	206	83.1
Extrapulmonary (243)	243	49.5
Category of TB		
New	454	92.5
Relapse	5	1.0
Failure	2	0.4
Default	3	0.6
Transfer in	19	3.9
Others^a^	8	1.6
HIV Status (291)		
Positive	82	28.2
Negative	209	71.8
Unknown	200	40.7

^a^Other: include loss to follow up

### Treatment outcomes

The treatment outcome of the 491 paediatrics TB patients assessed showed that the overall successful treatment (cure rate) was 420(85.5 %). The treatment success in males was 85.8 % while it was 84.9 % in females (*P* > 0.05). There was no significant differences in the treatment success of new TB cases compared to relapse cases which was 85.7 and 60 %, respectively (*P* = 0.32). The treatment success of HIV negative children was higher, 93.8 % compared to children with TB-HIV co-infection who had cure rate of 70.7 % (*P* =0.00). Similarly, children with unknown HIV serostatus had a higher treatment success of 82.5 % compared to the 70.7 % of the HIV positive cases (*P* = 0.00). Children in the age group 5–9 had higher treatment success of 123(88.5 %) compared to children of under 1 year of age 24(72.7 %) (*P* < 0.05). The treatment success was higher among patients with EPTB, (86.8 %) compared to those with PTB (81.0 % for SPPTB and 85.0 % of SNPTB).

The poor outcome of treatment identified showed that 3(0.6 %) were defaulters from treatment, 9(1.8 %) died, 2(0.4 %) were treatment failure and 55(11.2 %) were transferred out. The treatment outcome reports were unknown in 3(0.6 %) cases of the pediatric patients. The death rate is higher in the age range of 1–4 which was 3/74(4.1 %) followed by age group 10–14 with death rate of 4/245(1.6 %) (Table 3).

**Table 3:** Treatment outcomes, Age, Gender, Type of TB, Patient category and HIV status of children

Characteristics	Treatment outcomes						
	Success No (%)	Transfer out No (%)	Default No (%)	Death No (%)	Failure No (%)	Missing No (%)	Total No (%)
Gender							
Male	188 (85.8)	23 (10.5)	2 (0.9)	5 (2.3)	0 (0.0)	1 (0.5)	219 (44.6)
Female	232 (85.3)	32 (11.8)	1 (0.4)	4 (1.5)	2 (0.7)	1 (0.7)	272 (55.4)
Age group							
< 1	24 (72.7)	9 (27.3)	0 (0.0)	0 (0.0)	0 (0.0)	-	33 (6.7)
1–4	64 (86.5)	7 (9.5)	0 (0.0)	3 (4.1)	0 (0.0)	-	74 (15.1)
5–9	123 (88.5)	12 (8.6)	0 (0.0)	2 (1.4)	0 (0.0)	2 (1.4)	139 (28.3)
10–14	209 (85.3)	27 (11.0)	3 (1.2)	4 (1.6)	2 (0.8)	-	245 (49.9)
Type of TB							
SPPTB	34 (81.0)	4 (9.5)	0 (0.0)	2 (4.8)	1 (2.4)	1 (2.4)	42 (16.9)
SNPTB	175 (85.0)	25 (12.1)	1 (0.5)	3 (1.5)	1 (0.5)	1 (0.5)	206 (83.1)
EPTB	211 (86.8)	26 (10.7)	2 (0.8)	4 (1.6)	0 (0.0)	-	243 (49.5)
Category of TB							
New	389 (85.7)	48 (10.8)	3 (0.7)	8 (1.8)	2 (0.4)	4 (0.9)	454 (92.5)
Relapse	3 (60.0)	2 (40.0)	0 (0.0)	0 (0.0)	0 (0.0)	-	5 (1.0)
Failure	0 (0.0)	0 (0.0)	0 (0.0)	0 (0.0)	2 (0.4)	-	2 (0.4)
Transfer in	15 (78.9)	2 (10.5)	0 (0.0)	0 (0.0)	0 (0.0)	2 (10.5)	19 (3.9)
Others	4 (33.3)	4 (33.3)	0 (0.0)	1 (8.33)	0 (0.0)	2 (18.2)	11 (2.2)
HIV Status							
Positive	58 (70.7)	12 (14.6)	12 (14.6)	0 (0.0)	0 (0.0)	-	82 (28.2)
Negative	196 (93.8)	12 (5.7)	1 (0.5)	0 (0.0)	0 (0.0)	-	209 (71.8)
Unknown	165 (82.5	31 (15.5)	2 (1.0)	0 (0.0)	0 (0.0)	-	200 (40.7)

*SPPTB* Smear positive pulmonary tuberculosis, *SNPTB* smear negative pulmonary tuberculosis, *EPTB* extra-pulmonary tuberculosis

Missing: attended follow up irregularly

### Trend analysis of treatment outcomes in the 5 years

Majority of the study participants, 454(92.8 %) were new cases. The trend of the treatment outcome showed an increment in treatment success rate during the study period ranging from 78.0 % in 2009 to 92.6 % in 2013. The incidence of the disease slightly decreased across the years, except in 2010 which showed increment.

In the study, the rate of transfer out varied from 5(5.1 %) in 2009 to 20 (20.0 %) in 2012. Highest death rate of 4.1 % was observed in 2012 (Table 4).

**Table 4:** Trends of treatment outcomes and TB types in children in the five year period

Treatment outcome and type of TB	Time (in years)					Total No (%)
	2009 No (%)	2010 No (%)	2011 No (%)	2012 No (%)	2013 No (%)	
Treatment outcome						
Success (cured)	78 (78.0)	86 (85.1)	83 (84.7)	87 (88.8)	87 (92.6)	420 (85.5)
Transferred out	21 (21.0)	11 (10.9)	14 (14.3)	5 (5.1)	4 (5.3)	55 (11.2)
Default	0 (0.0)	2 (2.2)	0 (0.0)	1 (1.0)	0 (0.0)	3 (0.6)
Death	0 (0.0)	1 (1.0)	1 (1.0)	4 (4.1)	3 (3.2)	9 (1.8)
Failure	0 (0.0)	1 (1.0)	0 (0.0)	1 (1.0)	0 (0.0)	2 (0.4)
Unknown	1 (1.0)	0 (0.0)	0 (0.0)	0 (0.0)	0 (0.0)	2 (0.6)
Total	100 (20.4)	101 (20.6)	98 (19.9)	98 (19.9)	94 (19.1)	491 (100)
Type of TB						
SPPTB	9 (9.0)	10 (9.9)	9 (9.2)	7 (7.1)	7 (7.4)	42 (8.6)
SNPTB	42 (42.0)	41 (40.6)	38 (38.8)	44 (47.9)	41 (43.6)	206 (42.0)
EPTB	49 (49.0)	50 (49.5)	51 (52.0)	47 (48.0)	46 (48.9)	243 (49.5)

*SPPTB* smear positive pulmonary tuberculosis, *SNPTB* smear negative pulmonary tuberculosis, *EPTB* extra-pulmonary tuberculosis

**Fig. 1:**
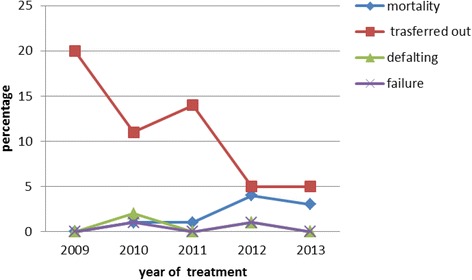
Trend of death, treatment defaulting, transferred out and unknown treatment outcome of pediatric TBs cases

**Fig. 2:**
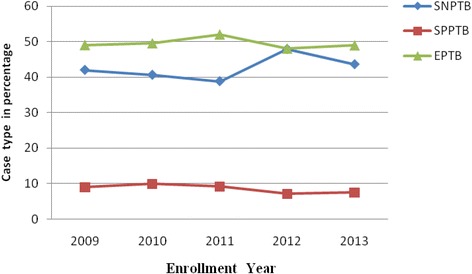
Trends of childhood tuberculosis types in the five years period

**Fig. 3:**
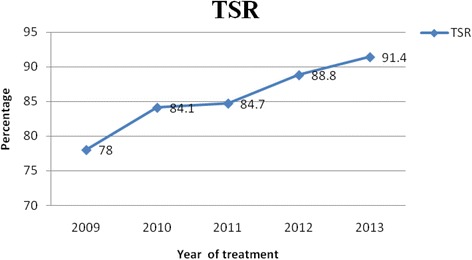
Trend of treatment success rate (TSR) of childhood tuberculosis in children treated in Zewditu Memorial hospital

The proportions of SPPTB was 42 (8.6 %), the highest being 9.9 % during 2010 (Table 4). The trend of EPTB cases varied across the study period from (52.0 %) in 2011 to (48.0 %) in 2012. The trend of unsatisfactory treatment outcomes during the study period (i.e. died, failed, defaulted and unknown treatment outcome) is presented in (Fig. 1). The assessment of TB types in the 5 years showed that the percent of EPTB cases ranged from 48 to 52 % compared to the SPPTB cases which ranged from 7.1 to 9.9 % and SNPTB cases from 38.8 to 47.9 % (Fig. 2). Treatment success rate consistently increased progressively from 78 % during 2009 to 91.4 % in 2013. The highest treatment success rate (TSR) of 91.4 % was observed in 2013 compared to TSRs across the earlier years (Fig. 3).

### Treatment outcome and associated factors

There was no significant difference in the treatment outcomes among male and female patients (*P* = 0.97). Children in the age group 5–9 years had statistically significant treatment success rate compared to the under 1 year age children (*P* = 0.04). On the other hand in multivariate adjusted model, variables like age, HIV status and pulmonary positive case types have shown significant association with the treatment success (Table 4). On multivariate logistic regression, children in the age group 5–9 years were independently associated with successful treatment outcomes [AOR = 0.59 (0.62–0.97), *P* = 0.04].

On the other hand, patients with SPPTB [AOR = 0.72 (0.53 – 0.92) and those with unknown serostatus [AOR = 0.94 (0.57 – 1.68)] had significantly lower treatment success rates.

Children who were HIV negative showed higher rate of treatment success compared to the HIV positive patients [AOR = 6.66 (3.07–14.47), *p* =0.00). The associated factors with TSRs were depicted in Table 5.

**Table 5:** Predictors of treatment outcome in Children with TB

Characteristics	Treatment outcome		Univariate analysis		Multivariate analysis	
	Successful No (%)	Unfavorable No (%)	COR (95 % CI)	*P* value	AOR (95 % CI)	*P* value
Gender						
Female	232 (85.3)	40 (15.1)	Referent			
Male	188 (86.1)	31 (13.9)	0.96 (0.56–1.64)	0.97	0.97 (0.97–1.67)	0.97
Age in years						
< 1	24 (72.7)	9 (27.3)	Referent			
1–4	64 (85.0)	10 (15.0)	0.43 (0.14–1.28)	0.15	0.64 (0.63–1.35)	0.15
5–9	123 (88.5)	16 (11.5)	0.35 (0.13–0.97)	0.05	0.59 (0.62–0.97)	0.04^a^
10–14	213 (87.1)	32 (12.9)	0.40 (0.16–1.02)	0.07	0.58 (0.56–1.03	0.06
Type of TB						
SPPTB	34 (81.0)	8 (19.0)	Referent			
SNPTB	175 (85.0)	31 (15.0)	0.66 (0.22–2.06)	0.66	0.77 (0.59–2.22)	0.68
EPTB	211 (86.8)	32 (13.2)	0.65 (0.26–1.66)	0.43	0.73 (0.96–1.74)	0.44
Category of TB						
New	389 (85.7)	65 (14.3)	Referent			
Retreatment	3 (60.0)	2 (40.0)	3.99 (0.46–30.03)	0.31	4.1 (0.49–30.31)	0.33
Transfer in	15 (83.1)	4 (16.9)	1.60 (0.43–5.35)	0.60	1.65 (0.49–5.60)	0.63
HIV status						
Negative	196 (93.8)	13 (6.2)	Referent			
Positive	58 (70.7)	24 (29.3)	6.24 (2.83–13.91)	*P* < 0.01	6.66 (3.07–14.47)	0.00^a^
Unknown	165 (82.5)	35 (17.0)	3.20 (1.57–6.61)	*P* < 0.01	3.44 (1.67–6.96)	0.00^a^

*COR* crude odds ratio, *AOR* adjusted odds ratio ^a^ Significant

## Discussion

As childhood TB reflects recent transmission, its burden provides an accurate measure of the level of TB in a community [15]. Treatment outcomes of TB in children are rarely evaluated by most TB programs in sub-Saharan Africa [16]. In 2007, the WHO has called for more studies to define the global epidemiology of childhood TB because the literature remains scant, dominated primarily by studies from industrialized countries [17]. Under 1 year-old children had the least involvement in 33 (6.7 %) while under 5 children constituted 107 (11.4 %) of the total TB patient population. This is similar to a study in India reporting 11 % despite the fact that rates of childhood TB are usually considered the highest among those aged 1–4 years [18]. Under-five children constituted the majority of childhood TB in previous studies in Africa [19] and Thailand [20]. The low number of under-1 year children with TB in this study could be due to missing of cases in investigation because of the diagnostic difficulties. In this study, older children in the age range of 10–14 years represented nearly half (49.9 %) of the cases with TB. This finding is similar to a report by Hailu et al. [14] who reported that 48.4 % of all the children assessed for TB were in the age range of 10–14 years.

TB was slightly higher in female children with male: female ratio of 0.8:1 (*P* > 0.05). This finding is in agreement with the previous study conducted in India, where high prevalence of 61.7 % was reported in females than in males [21]. However, many studies show similar infection rates in children unlike the predominance in adult males [22].

In the present study, nearly half of the cases, 243 (49.5 %) were due to EPTB, while the remaining 248 (50.5 %) were due to PTB. Of the PTB patients, 42 (16.9 %) had SPPTB and 206 (83.1 %) had SNPTB. The detection rate of *M. tuberculosis* (smear positivity) was slightly higher than a report by the WHO in which smear-positive TB in children aged <14 years accounted for 0.6–3.6 % [4]. However, our finding of SPPTB is similar with the16.8 % report by Tessema et al. [12]. However, the smear positivity rate is lower than a study done by Alavi et al. (2015) from Iran who reported a high rate of smear positive cases of 73 (72.3 %) [23].

In the present study, there was a clear trend of smear positivity in children with increasing age despite the overall prevalence EPTB children is higher. Majority of the children were <10 years old which is in agreement with previous studies [24]. However, the higher smear positivity was seen in older children. This could be due to the fact that young children are unable to produce sputum and thus have paucibacillary PTB. Thus, they are more likely to have EPTB than older children. In TB- endemic areas, the diagnosis of TB in children at lower age is mostly based on clinical and x-ray examinations. Thus, advanced TB diagnostic tools are critical for the study to avoid missing of childhood TB cases. Majority of the study participants, 454 (92.5 %) were new cases. This shows that the relapse and transferred in cases were small in number. HIV infection increases the susceptibility to TB. In this study, the prevalence of TB-HIV co-infection was 82 (28.2 %). Adejumo et al. [16] from Nigeria reported a 29 % co-infection and 27 % report from Thailand [20]. However, it is higher compared to previous reports of 10.9 % by Beza et al. (2013) from Ethiopia [25]. However, this is lower than a prevalence of 52 % reported by Fairlie et al. (2011) from South Africa [26].

The proportion of children with successful outcome is an indicator of the quality of TB case management [27]. In this study, the overall treatment success rate among all children with TB was 85.5 % which meets the WHO targeted of 85 % first set by the World Health Assembly in 1991 [1]. However, this finding was higher than in previous reports from southern Ethiopia where the treatment success rate was 49 % [28]. The proportions of treatment success were 85.7 % for new and 60 % for relapse (retreatment) cases for the entire study period (*P* < 0.05). Children under 1 year old had worst treatment outcome of 72.7 %. Similar finding was reported by Adejumo et al. from Nigeria [16]. Children less than 5 years old had a lower treatment success compared to those above 5 years (82.2 % versus 86.5 %, respectively). These are consistent with a study by Dangisso et al. [29].

Although the outcome of TB treatment in children is frequently not reported for various reasons some of the facts include lack of scientific studies, and the belief that childhood TB is not important factor for TB control [7]. In addition, socioeconomic conditions, malnutrition, over-crowding and HIV co-infection also contribute for the lower treatment outcomes [6]. This study found that the treatment outcome of childhood TB treated under DOTs program at Zewditu Memorial Hospital was satisfactory. The relatively higher success rate could be due to better case management with the availability of free TB treatment in the study health facilities. In addition, the low death rate of 1.8 % for all pediatric TB cases is lower than 5.8 % reported from Southern Ethiopia [8], 10.5 % reported from Botswana [30], 10.9 % from Tanzania [19] and 17 % from Malawi [31].

This study showed an overall default rate of 0.2 % among the pediatric TB patients in the study health facilities in the last 5 years. This is much lower compared to results from previous studies which showed high default rate in Ethiopia ranging from 3.8–20 % [32, 33].

Defaulter rate was low compared to a report in rural areas of South Ethiopia which was 13.9 % [34]. The mortality rate of 1.8 %) in our study consistent with a 3.3 % result reported by Hailu et al. [14] but lower than the rate 7.1 % reported by Salarri et al. from Iran [35]. Ethiopia is one of the highly affected countries by the TB/HIV co-epidemic. The WHO global report of 2008 estimated that in Ethiopia 40 % of TB patients tested for HIV were positive, while routine data from 2006/7 estimated that 31 % of TB patients were HIV positive [8]. In the present study, 82 (16.7 %) children had TB/HIV co-infection. This result is lower than the previous reports because of the reduced incidence of HIV infection in the country. Recent studies have reported that co-infection with HIV, young age, having smear-positive PTB, failing to convert to smear-negative after 2 months of treatment and living in a rural area were independent risk factors for unfavorable treatment outcome [7, 26]. Similarly, in the current study, co-infection with HIV, and age group of 5–9 year were risk factors for unfavorable outcome (Additional file 1). The higher incidence of serious forms of TB (such as meningitis or miliary TB), the immaturity of the immune system, delayed diagnosis due to the low sensitivity of diagnostic techniques and the higher prevalence of other adverse conditions such as malnutrition in young children have been reported as factors contributing towards poor treatment outcome at this age [5]. Few years back, Graham and his colleagues [36] reported that Malawian children treated for TB often had insufficient blood levels of medication, which could be due to several reasons including inappropriate drug dosage and lower absorption. Similarly, Young children who are unable, or unwilling to swallow large number of tablets each day contributing to poor treatment outcomes. Thus, child friendly formulations are the most critical need to treat TB [37].

The limitations of the study include many patients were excluded from the study due to incomplete records. Another limitation was the absence of microbiologic confirmation in most diagnosed patients.

## Conclusions

TB treatment success rate in the current study has similar to the first set WHO target of 85 % and is higher than that reported from other studies in the country and the region. This relatively higher success rate could be attributable to better case management with the availability of treatment in the study area. This study showed that the outcome of treatment varied with age, sex and type of TB. In order to further improve the treatment success rate, continuous follow up with frequent support of patients during treatment course and strengthen the recording system are strongly recommended.

## Abbreviations

aOR, adjusted odds ratio; HIV, human immunodeficiency virus; TB, tuberculosis; WHO, World Health Organization; DOTS, directly observed treatment- short course; MoH, ministry of health; PTB, pulmonary tuberculosis; EPTB, extra pulmonary tuberculosis; SPPTB, smear-positive pulmonary tuberculosis; SNPTB, smear-negative pulmonary tuberculosis

## Additional file

Additional file 1: Childhood TB in Addis Ababa, Data. (XLS 70 kb)
